# Coexistence of TSH-secreting adenoma and primary hypothyroidism: a case report and review of literature

**DOI:** 10.1186/s12902-023-01357-8

**Published:** 2023-05-23

**Authors:** Xiaolu Ren, Xu Wang, Ge Chen, Xiaohai Liu, Hongchuan Guo, Mingchu Li

**Affiliations:** 1 Department of Neurosurgery, Xuanwu Hospital, Capital Medical University, 45 Chang Chun St, Beijing, 100053 China; 2 International Neuroscience Institute (China-INI), Beijing, China; 3 Department of Neurosurgery and Laboratory of Neurosurgery, Lanzhou University Second Hospital, Lanzhou, China; 4 Institute of Neurology, Lanzhou University, Lanzhou, China

**Keywords:** Thyrotropin-secreting adenoma, Autoimmune hypothyroidism, Thyrotropin

## Abstract

**Background:**

Thyrotropin-secreting adenoma (TSHoma) is the least common type of pituitary adenoma, these patients often present with symptoms of hyperthyroidism. When TSHoma patients combined with autoimmune hypothyroidism, it is critically difficult to diagnose for the specific confusion in the results of thyroid function test.

**Case presentation:**

One middle-aged male patient was presented with a sellar tumor on cranial MRI for headache symptoms. After hospitalization, a significant increase in thyrotropin (TSH) was revealed by the endocrine tests, while free thyronine (FT3) and free thyroxine (FT4) decreased, and the diffuse destruction of thyroid gland was revealed by thyroid ultrasound. Based on the endocrine test results, the patient was diagnosed as autoimmune hypothyroidism. After the multidisciplinary discussion, the pituitary adenoma was removed by endoscopic transnasal surgery, until the tumor was completely excised, for which TSHoma was revealed by postoperative pathology. A significant decrease of TSH was revealed by the postoperative thyroid function tests, the treatment for autoimmune hypothyroidism was conducted. After 20 months of follow-up, the thyroid function of patient had been improved significantly.

**Conclusion:**

When the thyroid function test results of patients with TSHoma are difficult to interpret, the possibility of combined primary thyroid disease should be considered. TSHoma combined with autoimmune hypothyroidism is rare, which is difficult to diagnose. The multidisciplinary collaborative treatment could help to improve the outcomes of treatment.

## Background

Thyrotropin-secreting adenoma (TSHoma) is a complex and rare disease. These patients often present with significant symptoms of hyperthyroidism, whose optic nerve may be compressed, the loss of visual acuity and visual field defects could be caused by optic chiasm along with the growth of tumor. thyrotropin (TSH), free thyronine (FT3) and free thyroxine (FT4) was significantly elevated in the endocrine tests of patient. The current optimal treatment strategy is complete resection of the tumor, so that these patients can be biochemically cured, TSH could remain at extremely low level after surgery, hypothalamus-pituitary-thyroid axis function could return to normal function, and hyperthyroidism symptoms can be completely relieved [1]. The incidence of primary hypothyroidism exceeds 1% [2, 3], the patients could show as fatigue, loss of appetite and other symptoms, with decreased FT4 and increased TSH feedback in the endocrine tests. TSHoma combined with primary hypothyroidism is very rare. Its responsiveness to TSH is significantly decreased for the impaired secretory function caused by primary thyroid disease, with the low level of FT4. However, the endocrine activity of TSH adenoma could induce to the increase of TSH, as well as the negative feedback mechanism caused by the decrease of FT4, so that TSH could be abnormally elevated. It is difficult to diagnose correctly based on the thyroid function test results of these patients for its specific confusion. When pituitary adenoma is difficult to diagnose early, it may cause continuous growth and enlargement of tumor, which could induce to the compressive effects and increase the difficulty of treatment [4]. One rare case of TSHoma with primary hypothyroidism was reported in the study, who was successfully cured after multidisciplinary collaboration.

## Case presentation

**Fig. 1:**
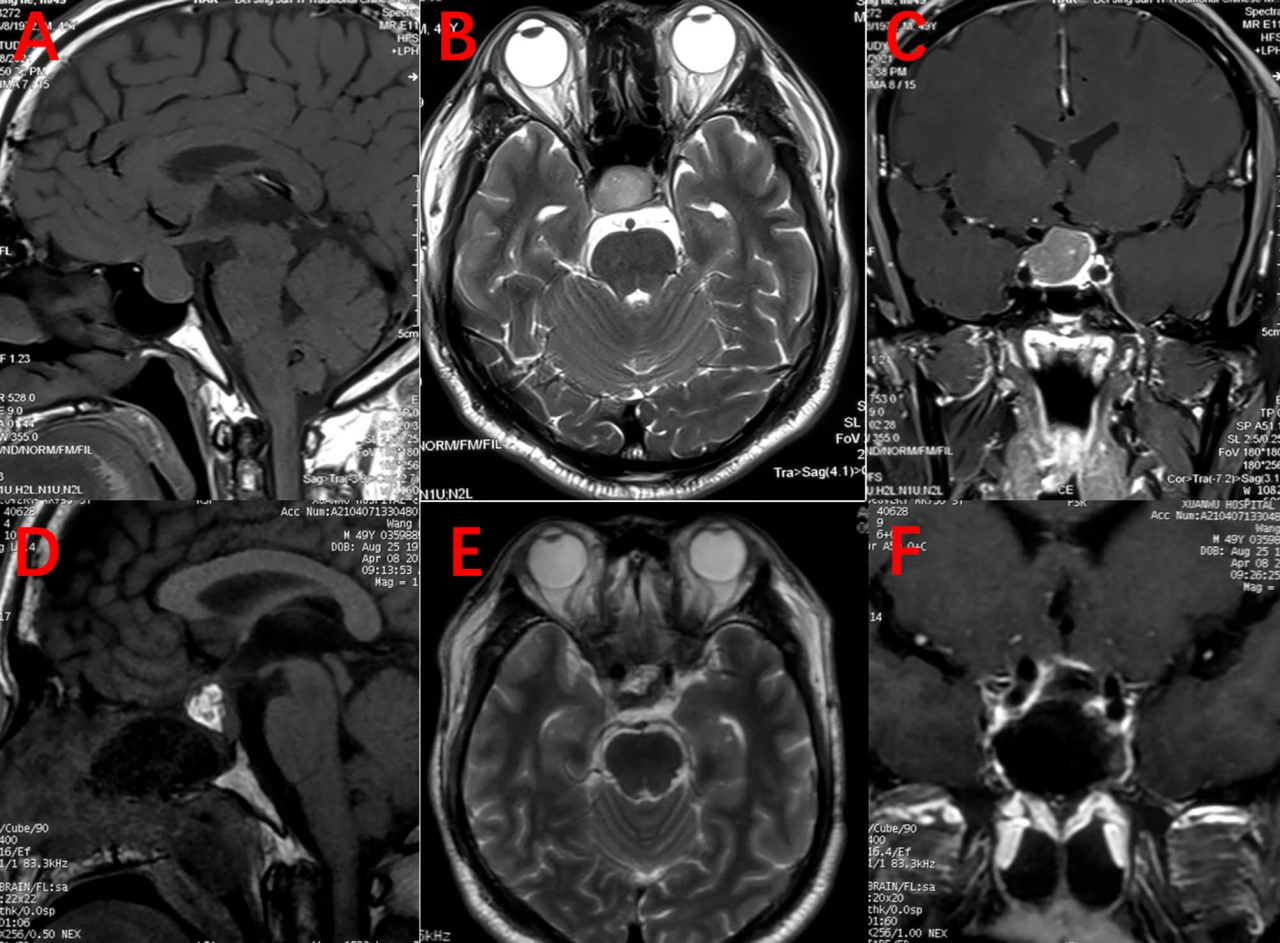
**A**-**C** was preoperative cranial MRI, the sellar tumor was visible, the optic chiasm was pushed upward by the tumor, and the normal pituitary gland was pushed to the left side. **D**-**F** was postoperative MRI, which observed that the tumor was completely excised, the autologous adipose tissue was packed in the sella turcica, and the normal pituitary gland was preserved

One 49-year-old male visited to the neurosurgical department for the intermittent headache lasting for 3 months. The cranial MRI revealed a sellar tumor, which reminded as pituitary adenoma (Fig. 1, A-C). Thyroid function tests were performed after the patient was admitted into Xuanwu Hospital Capital Medical University, which showed a significant increase in TSH, more than 150 uIU/ml (the normal range 0.34–5.5 uIU/m), FT3 and FT4 was lower than normal levels. Prolactin was slightly increased: 13.65 ng/ml (2.64–13.13 ng/ml). Because pituitary adenoma was clearly diagnosed while TSH was significantly elevated, TSHoma or central hypothyroidism caused by nonfunctioning pituitary adenomas was initially diagnosed. It was difficult to explain the decrease in FT3 and FT4 in TSHoma and central hypothyroidism, the multidisciplinary consultation was used and suspected that the patient had a primary disease of the thyroid gland. Thyroid ultrasonography showed diffuse destruction and nodular lesions of the thyroid gland. According to the endocrine findings and clinical manifestations, TSHoma with primary hypothyroidism was diagnosed. In addition, the prolactin of patient was slightly elevated, which may be caused by pituitary stalk effect. After multidisciplinary discussion, the treatment scheme was oral administration of levothyroxine (L-T4) at a dose of 75 ug daily, the tumor was excised by surgery until thyroid function was improved. After 4 weeks of oral administration of L-T4, the rechecked TSH was still very high (150 uIU/ml), then the tumor was removed by the transnasal endoscopic surgery, the boundary between the tumor and the normal pituitary gland was carefully identified during the operation, to protect the pituitary tissue, and remove the tumor in blocks and completely. After removing the tumor, the less leakage of cerebrospinal fluid was observed. Thus, the sellar floor was filled with a small amount of autologous fat to prevent the postoperative cerebrospinal fluid leakage. Postoperative re-examination of cranial MRI showed no residual tumor (Fig. 1, D-F), and re-examination of thyroid function was shown with a significant decrease in TSH. The histopathological result was pituitary adenoma, and immunohistochemistry findings were shown in Table 1. The patient recovered uneventfully without complications and his headache symptoms were gradually improved. Oral administration of L-T4 was taken at a dose of 75 ug daily and thyroid function was closely monitored. Re-examination of thyroid function 20 months after surgery showed that FT3 and FT4 were in the normal range, and TSH decreased to the normal range. Thyroid function tests before the surgery and after the surgery are shown in Table 2.

**Table 1:** The histopathological result and immunohistochemistry findings

LH	FSH	ACTH	PRL	TSH	GH	Pit-1	T-pit	CK18	P53	ER	CK	EMA	Syn	GFAP	SF-1
+	-	-	Little +	+	-	+	-	+	Little +	-	+	-	+	-	-

**Table 2:** Thyroid function tests before the surgery and after the surgery

	TSH (0.34–5.5 uIU/ml)	FT3 (2.3–4.2 pg/ml)	FT4 (0.89–1.76 ng/dl)	Prolactin (2.64-13.13ng/ml)
Pre-operation	150	2.26	0.49	13.65
Post-operation	20.3	1.79	1.03	1.49

## Discussion and conclusions

Patients with pituitary adenomas often have with thyroid dysfunction with various manifestations, for which the diagnosis and treatment are confusing. This patient should be differentiated from central hypothyroidism. Central hypothyroidism is very common in pituitary adenomas [5–7], for which the thyroid function tests could show as decreased FT4, decreased TSH or the normal level. In this case, FT3 and FT4 was lower than normal level, while TSH was abnormally elevated. Therefore, the patient should be evaluated as TSHoma. Fortunately, the sellar tumor of the patient was found early for his headache symptoms, the tumor did not grow to a large size, so that the surgical resection of the tumor was relatively simple. However, if primary thyroid disease is ignored, the long-term hypothyroidism may occur after surgery.

If the patient has is small tumor size, without any pituitary adenoma-related symptoms, the thyroid function test only is shown with a decrease in FT3 and FT4, and elevated TSH, it is easy to ignore the diagnosis of TSHoma, only diagnosed as primary hypothyroidism. Despite patients with primary hypothyroidism have the higher TSH level, while it is rarely at abnormally elevated levels. After the oral administration of L-T4, TSH returns to normal levels. For the patient combined with TSHoma, TSH does not return to the normal level after oral administration of L-T4. Besides, the pituitary gland may become abnormal in patients with primary hypothyroidism. If FT4 decreases, pituitary hyperplasia, nodule formation, and even tumor formation could be caused by a negative feedback mechanism of the hypothalamic-adenohypophysis-thyroid axis [8, 9].

N N Ghannam et al. suggested that primary hypothyroidism could induce to the proliferation of thyroid-stimulating cells and leads to the formation of TSHoma [10]. M F Langlois et al. reported a patient with autoimmune thyroiditis and TSHoma, the conclusion whether the tumor was secondary to primary hypothyroidism could not be drawn yet [11]. In this case, the diffuse destruction of the thyroid gland was observed on ultrasound, and cranial MRI showed that there was large pituitary adenoma of patient with 2 cm in diameter, and the sequential order of the tumor and primary hypothyroidism was difficult to confirm. Patients with hypothyroidism could present with skin changes [12, 13]. Our patient developed hair loss and coarse skin several years before surgery, but no pigmentation was shown.

In addition, prolactin was slightly elevated in this patient. In 30% of all TSHoma, the tumor could co-secrete TSH and other hermones, such as growth hormone and prolactin [14, 15]. The co-secretion of different kinds of hormone may be induced by the expression of common transcription factors by anterior pituitary cells. For the patient in the current study, the elevated level of prolactin may also be caused by pituitary stalk effect. Moreover, thyrotropin- releasing hormone elevation may be caused by the negative feedback mechanism, which could act on pituitary prolactin cells and result in a mild elevation of prolactin.

TSHoma is the least common type of pituitary tumor, accounting for only 0.5 − 3% [1, 16]. TSHoma patients generally have obvious symptoms of hyperthyroidism, including increased FT4, increased TSH or the normal level of them. It is easy to diagnose before the surgery, the postoperative pathological examination can help confirm the diagnosis. Surgical removal of the tumor is the best treatment. But beyond that, medical therapies including somatostatin analogs and dopamine agonists and radiation therapy are also useful [17–19]. The patients often have thyroid lesions caused by the effect of TSH on thyroid gland. The thyroid nodular lesion of this patient was seen in the thyroid ultrasound. There is very rare TSHoma with primary hypothyroidism. On account of self-damage of thyroid gland, decreased endocrine function, decreased FT3 and FT4, the excessive TSH secreted by TSHoma as well as primary hypothyroidism caused by negative feedback regulation, TSH was at abnormally elevated levels. Most of the current studies on TSHoma with primary hypothyroidism have been individual case reports [10, 11, 20, 21]. Primary hypothyroidism is often easily diagnosed in these patients, and pituitary adenomas could be covered up in the thyroid function test results. When the oral administration of L-T4 is difficult to reduce TSH level in patients with primary hypothyroidism, the onset of TSHoma should be considered, and cranial MRI should be performed early to prevent the neurological deficits caused by continuous growth of tumor.

## Conclusion

Although there is very rare primary hypothyroidism with TSHoma, the medical staffs should still be vigilant to treat hypothyroid patients with abnormally elevated TSH, the possibility of TSHoma should be considered. Multidisciplinary collaboration helps to achieve early diagnosis and treatment. The primary lesion of the thyroid gland cannot be neglected after the total resection of TSHoma, the long-term treatment with L-T4 and the close monitoring of thyroid function is required.

## Data Availability

All the data generated and/or analyzed during this study are included in this published article.

## Abbreviations

TSHoma: thyrotropin-secreting adenoma
TSH: thyrotropin
FT3: free thyronine
FT4: free thyroxine
L-T4: levothyroxine
